# CBP/β‐Catenin Signaling in Hepatocytes Plays Pivotal Roles in MMP‐7‐Mediated Liver Fibrosis in a Metabolic Dysfunction‐Associated Steatohepatitis Mouse Model

**DOI:** 10.1096/fj.202504510R

**Published:** 2026-03-12

**Authors:** Jun Imamura, Masamichi Kimura, Yumi Otoyama, Koji Nishikawa, Michinori Kohara, Kiminori Kimura

**Affiliations:** ^1^ Department of Hepatology Tokyo Metropolitan Cancer and Infectious Diseases Center Komagome Hospital Tokyo Japan; ^2^ Department of Microbiology and Cell Biology Tokyo Metropolitan Institute of Medical Science Tokyo Japan

**Keywords:** cAMP response element‐binding protein‐binding protein, MASH, MMP‐7, P300, Wnt/β‐catenin

## Abstract

In this study, we focused on CBP/β‐catenin signaling in hepatocytes and investigated its involvement in fibrosis in a metabolic dysfunction‐associated steatohepatitis (MASH) model using a choline‐deficient, L‐amino acid‐defined, high‐fat diet (CDAHFD). Alb‐Cre/CBPKO (CBP^hep‐KO^), Alb‐Cre/P300KO (P300^hep‐KO^), and Alb‐Cre mice were fed CDAHFD for 4 months, and liver tissue was used for Sirius Red staining. After 4 months of feeding, the CDAHFD CBP^hep‐KO^ mice showed a significant decrease in the liver fibrosis area compared to the other mice. RNA was extracted from the livers and analyzed for fibrosis‐related gene expression using RT‐PCR, PCR array, and RNA‐seq analysis. RNA‐seq analysis revealed a significant difference in MMP‐7 expression between the livers of CBP^hep‐KO^ and P300^hep‐KO^ mice. Next, MMP‐7KO and control mice were fed CDAHFD for 4 months. The MMP‐7KO mice showed a significant decrease in the liver fibrosis area. The C57BL/6 mice were fed CDAHFD, and 4 months later, AAV8/ShMMP‐7 and AAV8/control (2 × 10^12^ copies/mouse) were injected twice at 2‐week intervals, and fibrosis was assessed. The AAV8/ShMMP‐7 treatment reduced the fibrotic area and expression of *Col1A1* and *Col3A1* in the livers of CDAHFD‐fed mice. Thus, CBP/β‐catenin signaling in hepatocytes has been implicated in MASH fibrosis via MMP‐7, suggesting that MMP‐7 is a potential target for new anti‐fibrosis drugs.

## Graphical abstract

CDAHFD‐induced hepatic steatosis stimulates CBP/β‐catenin signaling in hepatocytes, leading to increased production of the downstream effector MMP‐7. Hepatocyte‐derived MMP‐7 contributes to fibrogenic responses and is associated with the progression of liver fibrosis.

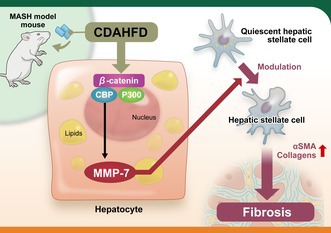

**Abbreviations**
AAV: adeno‐associated virus
ALT: alanine aminotransferase
CBP: cAMP response element‐binding protein‐binding protein
CDAHFD: choline‐deficient, L‐amino acid‐defined, high‐fat diet
DEGs: differentially expressed genes
FBS: fetal bovine serum
FPKM: fragments per kilobase of transcript per million mapped reads
GAN: Gubra‐Amylin NASH
HBV: Hepatitis B virus
HCV: hepatitis C virus
H&E: hematoxylin and eosin
HSC: hepatic stellate cell
MASH: metabolic dysfunction‐associated steatohepatitis
MMP: matrix metalloproteinase
PBC: primary biliary cholangitis
PCA: principal component analysis
TG: triglyceride

## 1 Introduction

Liver cirrhosis results from the abnormal accumulation of extracellular matrix during the repair of hepatocellular damage caused by factors such as hepatitis viruses, alcohol, and fatty deposits [1, 2]. The number of patients with metabolic dysfunction‐associated steatohepatitis (MASH) has rapidly increased with an increase in the obese population and is expected to increase further in the future [3, 4]. Fibrosis is an important prognostic factor in patients with MASH‐related cirrhosis, and the development of drugs to improve fibrosis is gaining momentum [5, 6]. Resmetirom (THR‐β agonist) was first approved by the Food and Drug Administration (FDA) in 2024 for the treatment of MASH [7, 8]. This is indicated for patients with MASH in the pre‐cirrhotic state, F2‐3 (F4 is cirrhosis). Its mechanism of action ameliorates fibrosis by improving fat metabolism in the liver, reducing fat accumulation, and suppressing inflammation rather than directly affecting fibrosis. Therefore, there are currently no drugs that target fibrosis in MASH cirrhosis, and the development of novel therapeutic agents is an urgent priority [9].

In organ fibrosis, fibroblasts/hepatic stellate cells (HSCs) become activated, leading to the excessive deposition of extracellular matrix components such as collagen [2, 10, 11]. In this process, persistent activation of the Wnt/β‐catenin pathway is suggested to exacerbate fibrosis through pathways including maintaining HSC activation, promoting fibroblast proliferation and anti‐apoptosis, cross‐talk with TGF‐β signaling, and enhancing the expression of ECM production genes [12, 13].

β‐catenin participates in transcription by binding to either of the coactivators CBP or P300. It has been reported that CBP/β‐catenin signaling is involved in the regeneration, proliferation, and maintenance of fibrosis, whereas P300/β‐catenin signaling promotes differentiation [14, 15, 16]. Therefore, inhibiting only the CBP/β‐catenin interaction is expected to suppress the activation of fibroblasts (activated HSCs) while preserving the p300/β‐catenin signaling pathway, thereby promoting tissue regeneration and differentiation [17, 18].

Recently, PRI‐724 was identified as a small‐molecule inhibitor of Wnt signaling that selectively disrupts the protein–protein interaction between β‐catenin and CREB‐binding protein [18, 19]. PRI‐724 has anti‐fibrotic effects in a bleomycin‐induced pulmonary fibrosis model and suppresses TGF‐β expression in the lungs [18]. Thus, Wnt signaling is involved in TGF‐β‐mediated fibrosis and has attracted attention as a target for anti‐fibrotic therapy [11]. We previously investigated whether PRI‐724 has antifibrotic effects in hepatitis C virus (HCV) transgenic mice and found a significant improvement in liver fibrosis [20]. We also reported the efficacy of PRI‐724 in other models of liver fibrosis, including carbon tetrachloride, bile duct, and MASH models, using a choline‐deficient, amino acid‐defined high‐fat diet (CDAHFD) [21, 22]. Based on the results of these nonclinical studies, clinical trials evaluating the safety and tolerability of PRI‐724 in patients with hepatitis B virus (HBV)/HCV‐related cirrhosis, primary biliary cholangitis, and hemophilia with HIV/HCV‐related cirrhosis have been completed, while their development is ongoing [23]. Although PRI‐724 inhibits the activation of hepatic stellate cells and induces the production of MMP‐8 and MMP‐9 in macrophages [20], the precise mechanism of fibrosis regression is unclear.

In this study, we generated Alb‐Cre/CBPKO (CBP^hep‐KO^) and Alb‐Cre/p300KO (P300^hep‐KO^) mice and fed them a CDAHFD to investigate the interaction between fibrosis progression and cAMP response element‐binding protein‐binding protein (CBP)/β‐catenin signaling in hepatocytes. Here, we demonstrated that liver fibrosis was suppressed in CBP^hep‐KO^ mice using the MASH model with CDAHFD and that MMP‐7 is involved as a downstream signal of CBP/β‐catenin. This study demonstrated that MMP‐7 is a promising therapeutic target for MASH.

## 2 Materials and Methods

### 2.1 Ethics Statement

All animal experiments were conducted according to the institutional guidelines of the National Academy of Sciences (Guide for the Care and Use of Laboratory Animals). The study protocol was approved by the Research Committee of the Tokyo Metropolitan Komagome Hospital (approval number 2). Ethical approval for human sample collection and analysis was obtained from the Ethics Committee of Tokyo Metropolitan Komagome Hospital (approval number 3200). Written informed consent was obtained from all participants before sample collection. This study was conducted in compliance with the Declaration of Helsinki and ARRIVE guidelines for reporting in vivo experiments.

### 2.2 Animals and Treatments

All mice were maintained in ventilated cages under 12 h light/dark cycles with free access to enrichment, water, and feed.

#### 2.2.1 CDAHFD Model

The following mouse strains were obtained from the Jackson Laboratory (Bar Harbor, ME, USA): albumin promoter‐driven Cre recombinase (Alb/Cre) mice [B6.FVB (129)‐Tg (Alb1‐cre)1Dlr/J, #016832], CBP^flox^ mice (B6.Cg‐*Crebbp* ^*tm1Jvd*^/J, #025178), and P300^flox^ mice (B6.129P2‐*Ep300* ^*tm2Pkb*^/J, #025168). CBP^flox^ and P300^flox^ mice were crossed with Alb/Cre mice to delete CBP or P300 from the hepatocytes. MMP‐7 KO mice (B6.129‐*Mmp7* ^*tm1Lmm*^/J, #005111) [24] were obtained from Jackson Laboratory. Eight‐week‐old specific pathogen‐free C57BL/6J male mice were purchased from CLEA Japan Inc. (Tokyo, Japan). Male mice were fed CDAHFD (A06071302; Research Diets Inc., New Brunswick, NJ, USA) for 16 weeks. The animals were then euthanized by exsanguination. Their livers were immediately removed, frozen in liquid nitrogen, and stored until further analysis. A portion of the dissected liver tissue was fixed in 10% formalin for histological analysis.

### 2.3 PRI‐724 Treatment Protocol

For pharmacological inhibition studies, PRI‐724 was administered intraperitoneally at 20 mg/kg body weight, two to three times per week starting at Week 12 of CDAHFD feeding. Mice were euthanized 4 weeks after treatment initiation for histological and molecular analyses.

### 2.4 Adeno‐Associated Virus (AAV) Transfection

AAVs (serotype 8) were custom‐packed in SignaGen Laboratories (Frederick, MD, USA). After designing an shRNA targeting mouse MMP‐7 (NM_010810) pAAV‐U6‐shRNA (mouse MMP‐7)‐CMV‐GFP‐WPRE‐polyA was constructed, and AAV was packaged. MMP‐7 shRNA (AAV8/shMMP‐7) (Cat # SL102806) or control shRNA (AAV8/control) (Cat # SL100862) was delivered via tail vein injection (2 × 10^12^ copies/mouse).

### 2.5 Cell Culture and Treatment

HepG2 cells (HB‐8065; ATCC, Manassas, VA, USA), a human hepatocellular carcinoma cell line, were cultured in DMEM (Gibco, Billings, MT, USA) supplemented with 1% penicillin–streptomycin and 10% fetal bovine serum (FBS). Cells were grown at 37°C under an atmosphere of 5% CO_2_ and 100% humidity for all experiments.

For steatosis induction, cells were treated with palmitate (Sigma‐Aldrich) for 24 h. Subsequently, the active form of PRI‐724 (C82) was added and incubated for an additional 24 h before RNA extraction [25]. The LX‐2 human hepatic stellate cell line (SCC064; Sigma‐Aldrich) was treated with recombinant human MMP‐7 (R&D Systems, 907‐MP) at concentrations ranging from 0.05–0.5 nM for 24 h prior to RNA extraction.

### 2.6 Immunofluorescence Staining

Frozen liver sections were fixed in 4% paraformaldehyde for 10 min, permeabilized with 0.3% Triton X‐100, and blocked using Blocking One Histo (Nacalai Tesque). Primary antibodies included: αSMA (Abcam, ab21027), CK19 (Cell Signaling Technology, 12434S), CBP (Santa Cruz Biotechnology, sc‐7300), and EP300 (GeneTex, GTX30614). Highly cross‐adsorbed donkey secondary antibodies conjugated to Alexa Fluor 488 or 647 were used. Nuclei were counterstained with DAPI. Images were acquired using a Keyence BZ‐X710 fluorescence microscope.

### 2.7 Serum Cytokines, Chemokines, and Parameters

Serum cytokine and chemokine levels were measured using Bio‐Plex Cytokine Assay Kits (Bio‐Rad Laboratories, Hercules, CA, USA), according to the manufacturer's instructions. Specifically, the Bio‐Plex Mouse Cytokine 40‐Plex Panel, Chemokine Panel, and MMP Panel were used. The samples were analyzed in a 96‐well plate reader using the Bio‐Plex Suspension Array System and Bio‐Plex Manager software (Bio‐Rad Laboratories). Human serum MMP‐7 was measured using the Human Total MMP‐7 ELISA Kit—Quantikine (R&D Systems Inc., Minneapolis, MN, USA). Serum alanine transaminase (ALT) levels were measured using the Transaminase CII test (Fujifilm Wako Pure Chemical Industries Ltd., Osaka, Japan).

### 2.8 Hydroxyproline and Triglyceride Measurement

A QuickZyme hydroxyproline assay (QuickZyme Bioscience, Leiden, the Netherlands) was used to quantify the hydroxyproline content in the liver. Liver tissues were homogenized and hydrolyzed in 6 N HCl at 110°C for 24 h. The absorbance of the samples was measured at 558 nm using a GloMax Explorer Multimode Microplate Reader (Promega, Madison, WI, USA). The hydroxyproline content was expressed as micrograms of hydroxyproline per gram of liver tissue.

Liver triglyceride content was measured using the LabAssay Triglyceride Kit (Fujifilm Wako Pure Chemical Industries Ltd.) according to the manufacturer's instructions.

### 2.9 RT‐qPCR

RNeasy and DNase Kits (Qiagen, Hilde, Germany) and a High‐Capacity cDNA Reverse Transcription Kit (Applied Biosystems, Waltham, MA, USA) were used for RNA extraction from liver tissues and cultured cells, DNA removal, and reverse transcription. qRT‐PCR was performed in triplicate using probes and primer sets purchased from Thermo Fisher Scientific and TaqPath qPCR Master Mix, CG (Applied Biosystems) in a LightCycler 480 (Roche Applied Science, Penzberg, Upper Bavaria, Germany). Gene expression was analyzed using TaqMan probes for *Col1a1* (Mm00801666_g1), *Col1a2* (Mm00483888_m1), *Col3a1* (Mm01254476_m1), *MMP‐7* (Mm00487724_m1), *αSMA* (Mm00725412_s1), and *Gapdh* (Mm99999915_g1). Target gene expression levels were normalized to those of *GAPDH* in each sample.

### 2.10 PCR Array

The RT^2^ Profiler PCR Array (Qiagen Sciences, Germantown, MD, USA) was used for mRNA expression analysis of WNT/β‐catenin‐related genes (mouse WNT signaling pathway, PAMM‐043Z and mouse WNT signaling targets, PAMM‐243Z) in the liver. The assay was performed using RT^2^ SYBR Green qPCR Master Mix and RT^2^ First Strand Kit (Qiagen). Target genes in the PCR arrays were obtained from the Qiagen website.

### 2.11 RNA Sequence

Total RNA was extracted from whole livers using RNeasy and DNase Kits (Qiagen). Library preparation, sequencing, quality control, read mapping, and gene expression quantification (fragments per kilobase of transcript per million mapped reads, FPKM) were performed by Novogene Inc. (Beijing, China). Briefly, mRNA was purified using poly T oligo‐attached magnetic beads, fragmented, and reverse‐transcribed using random hexamer primers. The quantified libraries were pooled and sequenced using an Illumina (San Diego, CA, USA) platform (NovaSeq 6000). Read mapping was conducted using HISAT2 (v2.0.5), which was selected for its ability to build a splice‐aware index based on gene model annotations, thereby enabling more accurate alignment to the reference genome. FeatureCounts (v1.5.0‐p3) was used to count the number of reads mapped to each gene, followed by the calculation of FPKM values based on the gene length and mapped read counts. Principal component analysis (PCA) was performed using R (v4.3.2), including genes with FPKM > 1 in at least three samples. Differentially expressed genes (DEGs) were identified using DESeq2 (v1.42.1). Gene Ontology annotation and enrichment analyses were performed using the enrichGO function from the clusterProfiler R package (v4.10.1).

### 2.12 Histological Analysis

Liver histology and immunohistochemistry were performed as described previously [26]. Mouse liver tissues were fixed in 10% formalin, sectioned, and stained with hematoxylin and eosin (H&E). Liver steatosis was assessed using Sudan IV staining. Collagen deposits were stained with Sirius Red (saturated picric acid containing 0.1% Direct Red 80 and 0.1% Fast Green FCF). In addition, the samples were immunohistochemically stained using antibodies against‐αSMA (Cell Signaling Technology, Danvers, MA, USA), F4/80 (Invitrogen), CBP (Cell Signaling Technology), and P300 (GeneTex Inc., Irvine, CA, USA) using the VECTASTAIN Elite ABC Kit or the M.O.M Immunodetection Kit (Vector Laboratories Inc., Burlingame, CA, USA).

Standardized computer‐assisted image analysis was performed to quantify Sirius Red‐positive areas. An independent pathologist blindly selected five Sirius Red‐stained parenchyma spots in all biopsy samples and automatically measured Sirius Red‐positive areas using HistoQuant software (3DHISTECH, Budapest, Hungary).

### 2.13 Human Liver Biopsy Specimens

Human liver biopsy specimens were obtained from patients with histologically confirmed nonalcoholic steatohepatitis (NASH) (*n* = 6) who underwent diagnostic liver biopsy at the Tokyo Metropolitan Cancer and Infectious Diseases Center Komagome Hospital. Normal liver tissues (*n* = 5) were obtained from nontumorous regions of liver specimens resected for benign or malignant liver tumors and were confirmed to be histologically normal.

All liver biopsy specimens were evaluated by experienced pathologists, and the diagnosis of NASH was established based on standard histological criteria, including steatosis, lobular inflammation, hepatocellular ballooning, and fibrosis stage. Formalin‐fixed and paraffin‐embedded liver tissue sections were used for immunohistochemical analyses. Immunostaining for CBP and P300 was performed to assess their expression patterns in hepatocytes. The number and distribution of CBP‐ or P300‐positive hepatocytes were compared between normal and NASH liver tissues.

### 2.14 Statistical Analysis

Data are expressed as the mean ± standard deviation (SD) of the data collected from at least three independent experiments. The means of the two groups were compared using a two‐tailed Student's *t*‐test, and the means of multiple groups were compared using one‐way ANOVA, followed by Bonferroni's post hoc tests using GraphPad Prism 10.0 (GraphPad Prism Inc., San Diego, CA, USA). Statistical significance was set at *p < 0.05; **p < 0.01; ***p < 0.005; ****p < 0.0001.

## 3 Results

### 3.1 CBP^hep^ ^‐KO^ but Not P300^hep‐KO^ Mice Exhibit Reduced Liver Steatosis and Fibrosis Under CDAHFD Feeding

**FIGURE 1:**
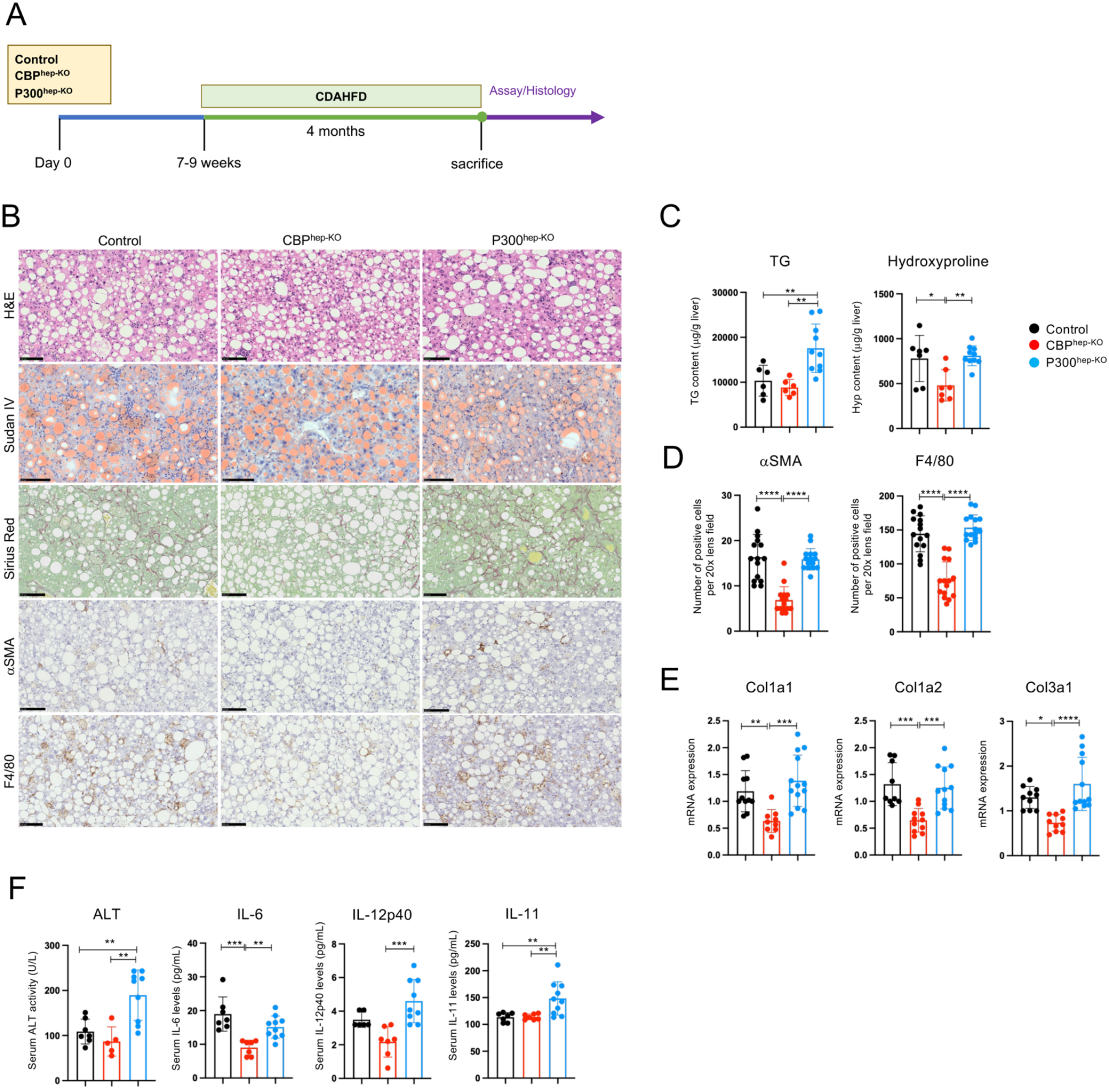
CBP^hep‐KO^ mice have reduced liver steatosis and fibrosis under CDAHFD feeding. Male control mice (8–10 weeks old, *n* = 7), CBP^hep‐KO^ mice (*n* = 6), and P300^hep‐KO^ mice (*n* = 10) were fed CDAHFD for 16 weeks. (A) Schematic representation of the treatment protocol. (B) Liver histology. H&E staining, Sudan IV staining, collagen deposition assessed by Sirius Red staining, and immunohistochemical staining using anti‐αSMA and F4/80‐antibodies (scale bars, 100 μm from the top figure). (C) Quantification of liver triglycerides and hydroxyproline. Liver tissue was analyzed using kits to quantify hepatic steatosis and fibrosis. (D) Immunohistochemical staining; the number of positive cells for hepatic stellate cells (αSMA) and macrophages (F4/80) in the liver was quantified. For each sample, the number of positive cells was counted in five fields at 20× magnification. (E) mRNA expression of the indicated genes in the livers as determined by RT‐qPCR. (F) Serum ALT, IL‐6, IL‐12p40, and IL‐11 levels (*n* = 5–10 per group). The results shown are representative of at least two independent experiments. Data are presented as mean ± SD. *p < 0.05; **p < 0.01; ***p < 0.005; ****p < 0.0001 by one‐way ANOVA.

To investigate CDAHFD‐induced liver steatosis and fibrosis, Alb‐Cre/CBPKO mice lacking hepatocyte‐specific CBP (CBP^hep‐KO^), its counterpart P300, Alb‐Cre/P300KO mice (P300^hep‐KO^), and Alb‐Cre mice (control) were fed a CDAHFD for 4 months, and the liver tissue was used for H&E, Sirius Red, Sudan IV, αSMA, and F4/80 staining. The treatment protocol is illustrated in Figure 1A. As shown in Figure 1B, after being fed CDAHFD, CBP^hep‐KO^ mice showed a significantly reduced fibrotic area, as measured by Sirius Red staining. In addition, hepatic steatosis, as measured by *triglyceride (TG)* quantification, and collagen deposition, as measured by hydroxyproline quantification, were significantly reduced in the CBP^hep‐KO^ mice (Figure 1C). In contrast, P300^hep‐KO^ mice showed exacerbated hepatic steatosis and fibrosis compared to CBP^hep‐KO^ mice.

Furthermore, immunostaining showed that the number of αSMA‐ and F4/80‐positive cells increased in P300^hep‐KO^ mice compared to that in CBP^hep‐KO^ mice, correlating with the degree of fibrosis and indicating an increase in hepatic stellate cells and macrophages. However, no differences in cell counts were observed between the control and P300^hep‐KO^ mice (Figure 1D).

Gene expression analysis of the liver revealed that the mRNA levels of *Col1a1*, *Col1a2*, and *Col3a1* were significantly reduced in the CBP^hep‐KO^ group compared to those in the control group (Figure 1E), whereas these mRNA levels were significantly higher in the P300^hep‐KO^ group than in the control group. Finally, the serum levels of ALT, IL‐6, IL‐12p40, and IL‐11 were significantly higher in P300^hep‐KO^ mice than in CBP^hep‐KO^ mice (Figure 1F).

### 3.2 The Intrahepatic Expression of MMP‐7 Was Markedly Reduced in CBP^hep^ ^‐KO^ Mice

**FIGURE 2:**
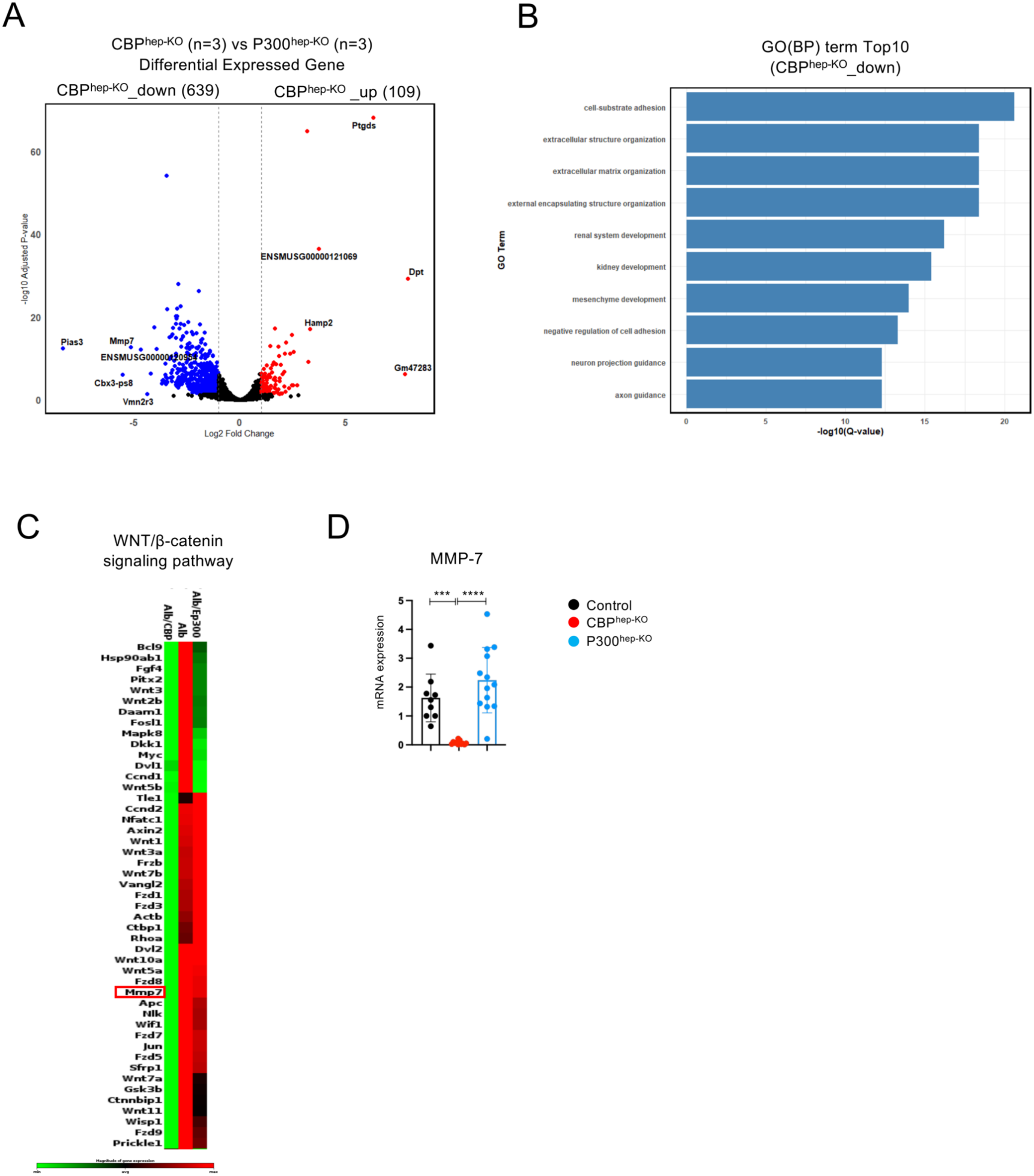
Intrahepatic expression of MMP‐7 is markedly reduced in CBP^hep‐KO^ mice. Male CBP^hep‐KO^ mice (8–10 weeks old, *n* = 3) and P300^hep‐KO^ mice (*n* = 3) were fed with a CDAHFD for 16 weeks. (A) Differentially expressed genes (DEGs). The volcano plot shows the results for the DEGs. (B) GOBP analysis of the top 10 downregulated genes in the livers of CBP^hep‐KO^ mice. (C) Clustergrams of PCR array analyses of WNT/β‐catenin‐related genes (WNT/β‐catenin signaling pathway). Green indicates low expression levels, while red indicates increased expression levels. (D) *MMP‐7* mRNA expression in the liver, as determined by RT‐qPCR (*n* = 9, 8, and 13 per group, respectively). The results shown are representative of at least three independent experiments. Data are presented as mean ± SD. *p < 0.05; **p < 0.01; ***p < 0.005; ****p < 0.0001, by one‐way ANOVA.

As mentioned above, we observed significant differences in steatosis and fibrosis in the liver tissues of the CBP^hep‐KO^ and P300^hep‐KO^ mice after CDAHFD feeding. Using this liver tissue, we first investigated the changes in gene expression in the liver using RNA‐seq. RNA was extracted from the liver tissues of control, CBP^hep‐KO^, and P300^hep‐KO^ mice (three mice each) after CDAHFD feeding, and RNA‐seq was performed. The volcano plot shows the results of the DEG analysis using liver samples from CBP^hep‐KO^ and P300^hep‐KO^ mice (Figure 2A). A total of 639 genes were downregulated in CBP^hep‐KO^ mice and 109 genes were upregulated. Among the numerous downregulated genes, we focused on MMP‐7, which was significantly differentially expressed in CBP^hep‐KO^ and P300^hep‐KO^ mice. Next, we performed a GOBP analysis of the top 10 most downregulated genes in the livers of CBP^hep‐KO^ mice. Among the top genes, we identified groups involved in fibrosis formation, such as cell‐substrate adhesion, extracellular structure organization, and extracellular matrix organization (Figure 2B). MMP‐7 was also identified in the RT‐PCR array based on the differences in gene expression between the two groups (Figure 2C). A significant decrease in MMP‐7 mRNA expression was confirmed by RT‐qPCR in several samples (Figure 2D).

### 3.3 MMP‐7KO Mice Exhibit Reduced Liver Steatosis and Fibrosis Under CDAHFD Feeding

**FIGURE 3:**
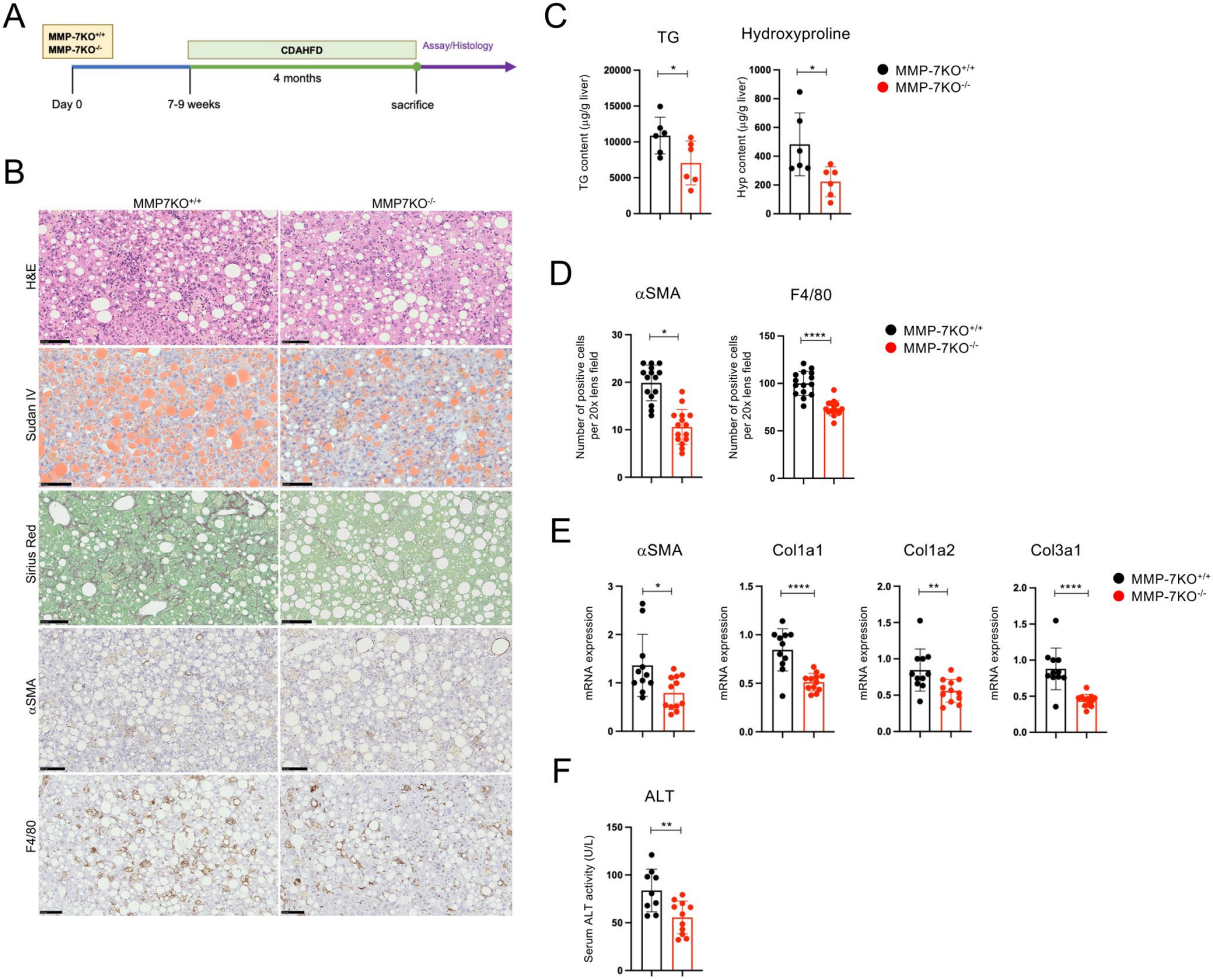
MMP‐7KO mice have reduced liver steatosis and fibrosis under CDAHFD feeding. Male MMP‐7KO^−/−^ and MMP‐7KO^+/+^ mice (8–10 weeks old, *n* = 6) were used as controls and fed CDAHFD for four months. (A) Schematic representation of the treatment protocol. (B) Liver histology. H&E staining, Sudan IV staining, collagen deposition assessed by Sirius Red staining, and immunohistochemical staining using anti‐αSMA and F4/80‐antibodies (scale bars, 100 μm from the top figure). (C) Quantification of liver triglycerides and hydroxyproline. Liver tissues were analyzed using kits to quantify hepatic fibrosis and steatosis. (D) Immunohistochemical staining: The number of cells positive for hepatic stellate cells (αSMA) and macrophages (F4/80) in the liver was quantified. For each sample, the number of positive cells was counted in five fields at 20× magnification. (E) mRNA expression of the indicated genes in the liver, as determined by RT‐qPCR. (F) Serum ALT levels (*n* = 8–10 per group). The results are representative of at least two independent experiments. Data are presented as mean ± SD. *p < 0.05; **p < 0.01; ***p < 0.005; ****p < 0.0001 by unpaired Student's *t*‐test.

MMP‐7 expression was significantly reduced in the livers of CBP^hep‐KO^ mice after CDAHFD feeding compared with that in P300^hep‐KO^ mice, prompting us to investigate the effect of MMP‐7 on the MASH fibrosis model. Eight‐week‐old male MMP‐7KO^−/−^ and MMP‐7KO^+/+^ control mice were fed a CDAHFD for 4 months (Figure 3A). As shown in Figure 3B, the Sirius Red‐positive area increased in control mice, and fibrosis was observed in the liver after CDAHFD. In contrast, the fibrotic region decreased in MMP‐7KO^−/−^ mice (Figure 3B). The same findings were observed in the quantitative results of hydroxyproline levels in the liver (Figure 3C). Liver tissues from MMP‐7 KO^−/−^ mice showed reduced lipid accumulation after CDAHFD feeding. The quantitative results of TG in the liver also showed that liver steatosis was reduced in MMP‐7 KO^−/−^ mice compared to that in control mice. We also found a significant reduction in *αSMA*, *Col1a1, Col1a2*, and *Col3a1* mRNA levels in the livers of MMP‐7 KO^−/−^ mice.

In addition, hepatocellular injury (ALT) was attenuated in MMP‐7KO^−/−^ mice (Figure 3F).

### 3.4 AAV8/shMMP‐7 Administration Inhibits CDAHFD‐Induced Fibrosis

**FIGURE 4:**
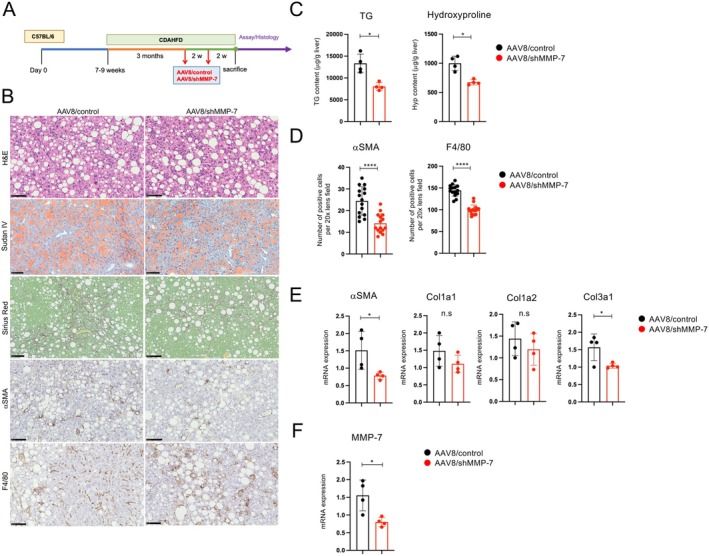
AAV8/shMMP‐7 administration inhibits CDAHFD‐induced fibrosis. Male C57BL/6 mice aged 8–10 weeks were fed CDAHFD for three months and then administered AAV8/control (*n* = 4) and AAV8/shMMP‐7 (*n* = 4) intravenously every two weeks. Liver tissue was analyzed four months after the start of feeding. (A) Schematic representation of the treatment protocol. (B) Liver histology. H&E staining, Sudan IV staining, collagen deposition assessed by Sirius Red staining, and immunohistochemical staining using anti‐αSMA and F4/80‐antibodies (scale bars, 100 μm from the top figure). (C) Quantification of liver triglycerides and hydroxyproline. Liver tissues were analyzed using kits to quantify hepatic steatosis and fibrosis. (D) Immunohistochemical staining: The number of cells positive for hepatic stellate cells (αSMA) and macrophages (F4/80) in the liver was counted. For each sample, the number of positive cells was counted in five fields at 20× magnification. (E, F) mRNA expression of the indicated genes in the liver, as determined by RT‐qPCR (*n* = 4 per group). Data represent the mean ± SD. *p < 0.05; **p < 0.01; ***p < 0.005; ****p < 0.0001 by unpaired Student's *t*‐test.

MMP‐7 KO^−/−^ mice exhibited reduced liver fibrosis and steatosis following CDAHFD administration. To determine whether hepatocytic MMP‐7 plays an important role in CDAHFD‐induced liver fibrosis, an adeno‐associated virus serotype 8 (AAV8)/shMMP‐7 was generated. Male C57BL/6 mice aged 8–10 weeks were fed CDAHFD for 3 months and intravenously administered AAV8/control (*n* = 4) and AAV8/shMMP‐7 (*n* = 4) every 2 weeks. Liver tissue was analyzed 4 months after the start of feeding (Figure 4A).

In the AAV8/control group, a fibrotic area positive for Sirius Red staining was observed, whereas in the AAV8/shMMP‐7 group, a reduction in the fibrotic area was observed (Figure 4B). The improvement in hepatic steatosis and fibrotic area in the liver tissue following AAV8/shMMP‐7 administration was also confirmed by quantitative analysis of TG and hydroxyproline levels in the liver tissue (Figure 4C). Furthermore, a significant decrease in the number of αSMA and F4/80‐positive cells was observed in the AAV8/shMMP‐7 group (Figure 4D). Gene expression analysis of RNA extracted from the liver revealed a significant decrease in *αSMA* and *Col3a1* expression following AAV8/shMMP‐7 administration (Figure 4E). These results suggest that MMP‐7 plays a critical role in CDAHFD‐induced liver fibrosis in hepatocytes. AAV8/shMMP‐7 injection significantly reduced MMP‐7 expression in the liver compared to AAV8/control injection, indicating that AAV8/shMMP‐7 was efficiently taken up by hepatocytes and suppressed MMP‐7 mRNA expression (Figure 4F).

### 3.5 Effects of MMP‐7 on Hepatic Stellate Cells

**FIGURE 5:**
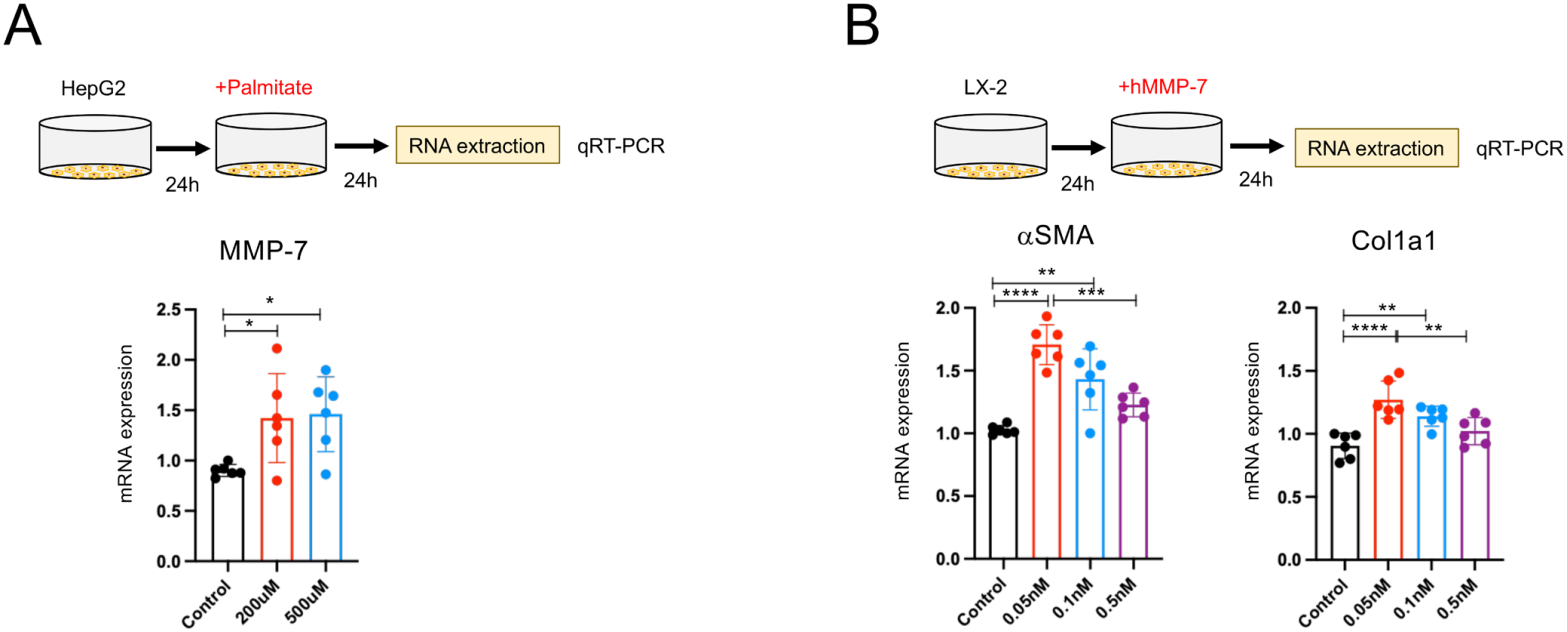
Effects of MMP‐7 on hepatic stellate cells. (A) Schematic of the treatment protocol. HepG2 cells were treated with palmitate (200 μM, 500 μM) for 24 h. The cells were then harvested, RNA was extracted, and MMP‐7 expression was analyzed using RT‐PCR. (B) Schematic representation of the treatment protocol. After culturing LX‐2 cells for 24 h, human recombinant MMP‐7 was added at concentrations ranging from 0.05 to 0.5 nM. Cells were harvested after 24 h, RNA was extracted, and the expression of αSMA and Col1a1 was analyzed by rt‐PCR. The results are representative of at least three independent experiments. Data represent the mean ± SD. *p < 0.05; **p < 0.01; ***p < 0.005; ****p < 0.0001, by one‐way ANOVA.

Hepatocyte‐derived MMP‐7 may contribute to fibrosis in a CDAHFD‐fed MASH model. We examined whether MMP‐7 expression was induced by hepatic lipid accumulation using an in vitro fatty liver model with palmitate [25]. HepG2 cells were treated with palmitate for 24 h, harvested, RNA was extracted, and MMP‐7 expression was analyzed using RT‐PCR. As shown in Figure 5A, MMP‐7 expression significantly increased in palmitate‐treated HepG2 cells, indicating that hepatocytes produce MMP‐7 in response to lipid accumulation.

Next, we examined the effect of MMP‐7 on a hepatic stellate cell line (LX‐2). After culturing LX‐2 cells for 24 h, human recombinant MMP‐7 was added at concentrations of 0.05, 0.1, and 0.5 nM. Cells were harvested after an additional 24 h for RNA extraction, and the expression levels of αSMA and Col1a1 were analyzed using RT‐PCR. As shown in Figure 5B, recombinant MMP‐7 altered the expression of fibrogenic markers in LX‐2 cells in a manner dependent on its concentration. The lowest concentration (0.05 nM) was associated with the greatest increase in αSMA expression, whereas higher concentrations (0.1–0.5 nM) showed an attenuated response, although the expression levels remained above the baseline. A similar trend was observed for Col1a1 expression levels.

These findings suggest that MMP‐7 directly influences the stellate cell phenotype and fibrogenic gene expression, supporting the role of hepatocyte‐derived MMP‐7 in regulating fibrogenic responses. Inhibition of CBP/β‐catenin signaling using the active form of PRI‐724 (C82) suppressed MMP‐7 expression in palmitate‐treated HepG2 cells, further supporting the role of CBP‐dependent transcription in regulating MMP‐7 production (Figure S1).

### 3.6 Serum MMP‐7 Levels Are Increased in Patients With MASH

**FIGURE 6:**
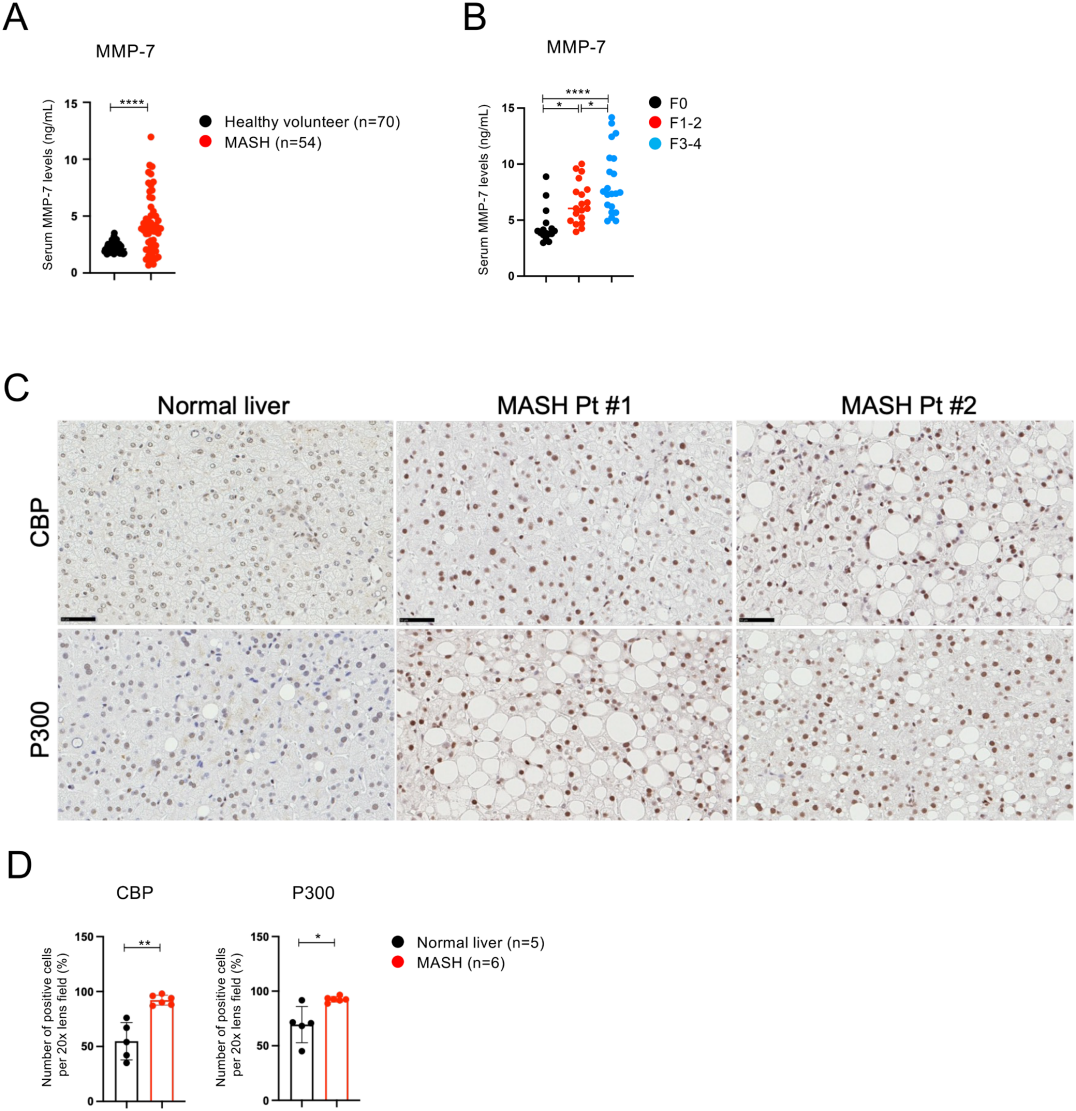
Serum MMP‐7 levels are increased in patients with MASH. (A) MMP‐7 concentrations were measured in serum samples from patients with MASH (*n* = 54) and healthy volunteers (*n* = 70). (B) MMP‐7 levels were measured in serum samples from patients with MASLD or MASH who underwent liver biopsy and had their fibrosis grade determined. F0 (*n* = 16), F1‐2 (*n* = 20), and F3‐4 (*n* = 22). (C) Representative immunohistochemical images of CBP and P300 in normal (*n* = 5) and NASH (*n* = 6) liver biopsy specimens. Increased nuclear CBP staining was observed in hepatocytes from NASH livers. The number of cells positive for CBP and P300 in the liver was determined. For each sample, the number of positive cells was counted in five fields at 20× magnification. (D) Quantification of CBP‐positive and P300‐positive hepatocytes in liver biopsy specimens. The number of positive cells was counted in five fields at 20× magnification.

As we found that MMP‐7 plays a crucial role in CDAHFD‐induced liver fibrosis, we compared serum MMP‐7 levels in patients with MASH (*n* = 54) and healthy volunteers (*n* = 70) as controls. As shown in Figure 6A, serum MMP‐7 levels were significantly higher in patients with MASH than in healthy volunteers. Next, we analyzed serum samples with known fibrosis stages to compare the F1‐2 and F3‐4 groups. Serum MMP‐7 levels were measured in patients with MASLD or MASH who underwent liver biopsy and fibrosis grades were determined [F0 (*n* = 16), F1‐2 (*n* = 20), and F3‐4 (*n* = 22)]. Serum MMP‐7 levels increased significantly with fibrosis stage, suggesting its potential usefulness as a diagnostic marker for fibrosis progression (Figure 6B).

To further investigate whether dysregulation of Wnt/β‐catenin–dependent transcription occurs in human diseases, we examined liver biopsy specimens obtained from patients with histologically confirmed NASH and compared them with normal liver tissues. Immunohistochemical staining was performed to evaluate the expression patterns of β‐catenin coactivators CBP and P300 in hepatocytes.

In normal liver tissues, CBP and P300 expression was low and limited to a small fraction of hepatocytes. In contrast, MASH liver biopsy specimens exhibited a marked increase in the number of hepatocytes positive for both CBP and P300, accompanied by enhanced nuclear localization, suggesting the global activation of β‐catenin–associated transcriptional machinery in diseased hepatocytes.

Importantly, although both coactivators were upregulated, CBP and P300 differentially regulate β‐catenin–mediated transcriptional programs. CBP‐associated β‐catenin signaling has been linked to injury responses and fibrogenic gene expression, whereas P300‐associated signaling is more closely related to differentiation and homeostatic transcriptional outputs [19, 27]. In this context, the observed increase in CBP‐positive hepatocytes in MASH livers provides clinical support for the notion that the upregulation of MMP‐7 in patients with MASH reflects, at least in part, enhanced CBP/β‐catenin–dependent transcriptional activity, even in the presence of concurrent P300 upregulation.

These findings are consistent with our experimental observations in mouse MASH models and support the translational relevance of the CBP/β‐catenin–MMP‐7 axis in human disease. Although additional regulatory mechanisms and contributions from nonparenchymal cell populations cannot be excluded, human biopsy data strengthen the link between dysregulated β‐catenin co‐activator signaling and MMP‐7–associated fibrogenic responses in NASH.

## 4 Discussion

We have previously reported that PRI‐724, an inhibitor of CBP/β‐catenin, is effective in several liver fibrosis models [21, 28]. Based on these preclinical results, we conducted clinical trials of PRI‐724 in humans with cirrhosis [23, 29]. We administered PRI‐724 twice weekly for 12 weeks to patients with HBV‐ and HCV‐related liver cirrhosis. Although we did not confirm an improvement in liver tissue fibrosis, we reported significant improvements in albumin levels and liver stiffness [23]. These results were also confirmed in a small number of patients with primary biliary cholangitis (PBC) [30] and hemophilia with HIV/HCV‐associated cirrhosis, suggesting the therapeutic potential of PRI‐724 in liver fibrosis [31]. However, the mechanism of action of PRI‐724 remains poorly understood. Therefore, we investigated the effects using CBP^hep‐KO^ mice and P300^hep‐KO^ in a CDAHFD‐induced MASH model. As expected, the CBP^hep‐KO^ mice exhibited suppressed liver fibrosis in the CDAHFD‐induced MASH model. Conversely, P300^hep‐KO^ mice exhibited marked worsening of liver fibrosis and significant hepatic steatosis, accompanied by elevated serum ALT levels, indicating liver dysfunction and increased macrophage infiltration. After approximately 6 months of CDAHFD feeding, liver fibrosis remained suppressed in CBP^hep‐KO^ mice, whereas P300^hep‐KO^ mice developed liver tumors. This revealed that differences in CBP/P300 signaling induced distinct pathological changes (data not shown). Based on these results, RNA‐seq was performed on liver tissue from CBP^hep‐KO^ and P300^hep‐KO^ mice to explore downstream signals of CBP/P300‐β‐catenin. Multiple genes were identified; however, MMP‐7, which showed a marked difference in expression, was the focus of this study. MMP‐7 expression was significantly suppressed in the livers of CBP^hep‐KO^ mice compared to that in control and P300^hep‐KO^ mice.

MMP‐7 is a zinc‐ and calcium‐dependent endopeptidase that degrades a wide range of ECM substrates [32]. MMP‐7 is a downstream target gene of Wnt/β‐catenin signaling and is involved in renal fibrosis via β‐catenin signaling [33, 34]. MMP‐7 serves as a noninvasive biomarker and is an important pathogenic mediator of kidney fibrosis [35]. Numerous reports have indicated the importance of MMP‐7 as a diagnostic marker for biliary atresia [36, 37, 38]. Furthermore, serum MMP‐7 levels are elevated in patients with NASH [39]. Reports indicating that MMP‐7 is positioned downstream of CBP/β‐catenin signaling originate from in vitro systems using lung cancer cells, as demonstrated using ICG‐001, an inhibitor of CBP/β‐catenin similar to PRI‐724 [40, 41].

In this study, we demonstrated in vitro that palmitate‐induced hepatic steatosis (palmitate) stimulates MMP‐7 production in hepatocytes and that hepatocyte‐derived MMP‐7 can modulate stellate cell activation and fibrogenic responses (Figure 5). Interestingly, recombinant MMP‐7 exerted a reproducible concentration‐dependent effect on LX‐2 cells, with the lowest dose (0.05 nM) producing the greatest increase in αSMA expression, and higher concentrations showing an attenuated, yet still above‐baseline, response. This pattern suggests that MMP‐7 may regulate the stellate cell phenotype in a context‐ and concentration‐dependent manner, rather than acting as a simple linear activator.

Furthermore, using a mouse model of MASH, we demonstrated the importance of hepatocyte‐derived MMP‐7 by generating AAV8/shMMP‐7. However, this study had several limitations. One limitation is the use of the CDAHFD model as a MASH model. CDAHFD is considered a lean MASH model that differs from obese MASH models. Nevertheless, it readily induces insulin resistance, inflammation, and fibrosis, making it useful for evaluating the therapeutic effects of fibrosis [42, 43]. Future research should focus on examining obesity‐type MASH models, such as the Gubra‐Amylin NASH (GAN) model [44].

Furthermore, we did not investigate other fibrosis models, particularly those involving cholestatic fibrosis, in our MASH‐induced liver fibrosis model. Given that MMP‐7 is involved in the pathogenesis of biliary atresia [38] and its expression increases in hepatocytes and cholangiocytes in liver injury caused by cholestasis [45], the therapeutic effect of MMP‐7 appears promising, even in a fibrosis model induced by cholestasis. Our previous study also confirmed significantly higher MMP‐7 expression in the liver of the biliary ligation model [28].

Although the present study was designed to investigate a hepatocyte‐centered CBP/β‐catenin–MMP‐7 axis using Alb‐Cre–based conditional deletion, it is important to acknowledge that Alb‐Cre–mediated recombination is not strictly restricted to hepatocytes and may occur in cholangiocytes under certain pathological conditions. Indeed, our coimmunofluorescence analyses demonstrated that while CBP and P300 expression was predominantly detected in ALB‐positive hepatocytes, a subset of CK19‐positive cholangiocytes also retained CBP and P300 expression, indicating an incomplete cell‐type specificity. Although Alb‐Cre–based conditional deletion was used to preferentially target hepatocytes, we acknowledge that recombination is not strictly restricted to hepatocytes and may occur in cholangiocytes under pathological conditions (Figure S2).

In addition to hepatocytes, cholangiocytes exhibit aberrant activation of Wnt/β‐catenin signaling during liver injury and are a major source of MMP‐7, particularly in cholestatic and ductular reaction–associated liver diseases. Large‐scale proteomic and clinical studies have identified MMP‐7 as a sentinel marker of cholangiocyte injury, with robust upregulation in biliary atresia and other cholestatic conditions, where MMP‐7 expression is localized predominantly to the injured bile duct epithelium [36, 38, 45, 46]. Furthermore, experimental cholestatic fibrosis models have demonstrated marked induction of hepatic MMP‐7 expression, and inhibition of CBP/β‐catenin signaling using PRI‐724 ameliorates fibrosis in these settings [28].

Given that PRI‐724 is a systemic inhibitor of CBP/β‐catenin interactions and not hepatocyte‐specific, its anti‐fibrotic effects may extend beyond hepatocytes to include cholangiocytes and other epithelial cell populations [21]. Consistent with this notion, the administration of PRI‐724 in a dietary MASH model resulted in significant attenuation of liver fibrosis, as shown in Figure S3, supporting the concept that the global inhibition of CBP/β‐catenin signaling exerts anti‐fibrotic effects in vivo. While the present study focused on hepatocyte‐derived MMP‐7 using Alb‐Cre–based genetic approaches and AAV8‐mediated knockdown strategies, these findings raise the possibility that the suppression of CBP/β‐catenin–dependent MMP‐7 signaling in cholangiocytes may also contribute to fibrosis attenuation. Future studies employing cholangiocyte‐specific genetic models, spatial transcriptomics, and coculture systems are necessary to dissect the cell‐type‐specific and intercellular signaling mechanisms underlying MMP‐7–mediated fibrosis progression in MASH.

Finally, we demonstrated that serum MMP‐7 concentrations in patients with MASH were significantly higher than those in healthy volunteers. While this finding has been reported in several studies, its correlation with fibrosis progression remains inconclusive owing to conflicting results [39, 47]. In the present study, we selected and examined patients diagnosed with MASH via liver biopsy and pathologically assessed at stages F0‐4. Serum MMP‐7 levels increased in accordance with the degree of fibrosis progression, suggesting its potential value as a diagnostic marker. Moving forward, rather than relying solely on MMP‐7, it is necessary to combine it with other parameters to enhance diagnostic accuracy.

In summary, we demonstrated that CBP^hep‐KO^ mice had significantly reduced liver steatosis and fibrosis compared to control and P300^hep‐KO^ mice in the CDAHFD‐fed MASH mouse model. We confirmed that MMP‐7, a downstream signal of CBP/β‐catenin, was significantly downregulated in the livers of CBP^hep‐KO^ mice. Consequently, when we generated a CDAHFD‐fed MASH mouse model using MMP‐7 KO^−/−^ mice, liver steatosis and fibrosis were suppressed. These results were reproducibly confirmed by administering AAV8/shRNAMMP‐7, which specifically knocked down MMP‐7 in hepatocytes. These findings suggest that MMP‐7 is a potential therapeutic target for MASH liver fibrosis and may also serve as a diagnostic marker. Taken together, our results support a hepatocyte‐centered CBP/β‐catenin–MMP‐7 axis as a regulatory mechanism contributing to the progression of fibrosis in MASH.

## Author Contributions

K.K. received the research funding and designed the research and experimental plans. J.I., M.K., Y.O, and K.N. performed the experiments and analyzed the data. M.K. supervised the study. J.I., M.K., Y.O., and K.K. performed the experiments. J.I. and K.K. interpreted the data and wrote the manuscript. All authors approved the final version of the manuscript.

## Funding

This work was supported by grant numbers 18pc0101024s0101, 20ek0109457h0001, and 20lm0203057h0003 from the Japan Agency for Medical Research and Development (AMED) and the Program for the Promotion of Fundamental Studies in Liver Cirrhosis of the Tokyo Metropolitan Government (to K.K.).

## Conflicts of Interest

The authors declare no conflicts of interest.

## Supporting information


**Figure S1:** fsb271685‐sup‐0001‐FigureS1‐S3.pdf.

## Data Availability

The data supporting the findings of this study are available in Sections 2 and 3.
