# Supplementary figures and images for: CBP/β‐Catenin Signaling in Hepatocytes Plays Pivotal Roles in MMP‐7‐Mediated Liver Fibrosis in a Metabolic Dysfunction‐Associated Steatohepatitis Mouse Model
Source: FASEB J. 2026 Mar 12;40(6):e71685. doi: 10.1096/fj.202504510R (PMC12980565; doi:10.1096/fj.202504510R)

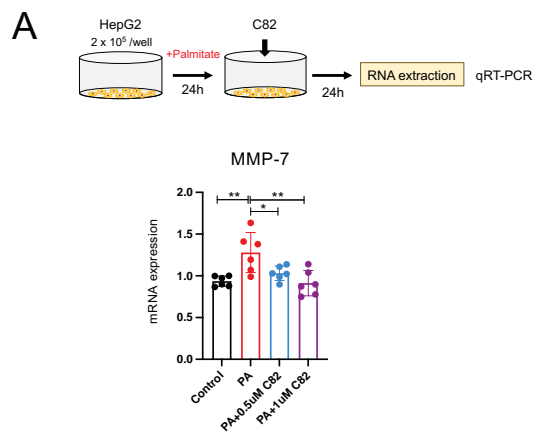

Supplementary Figure S1

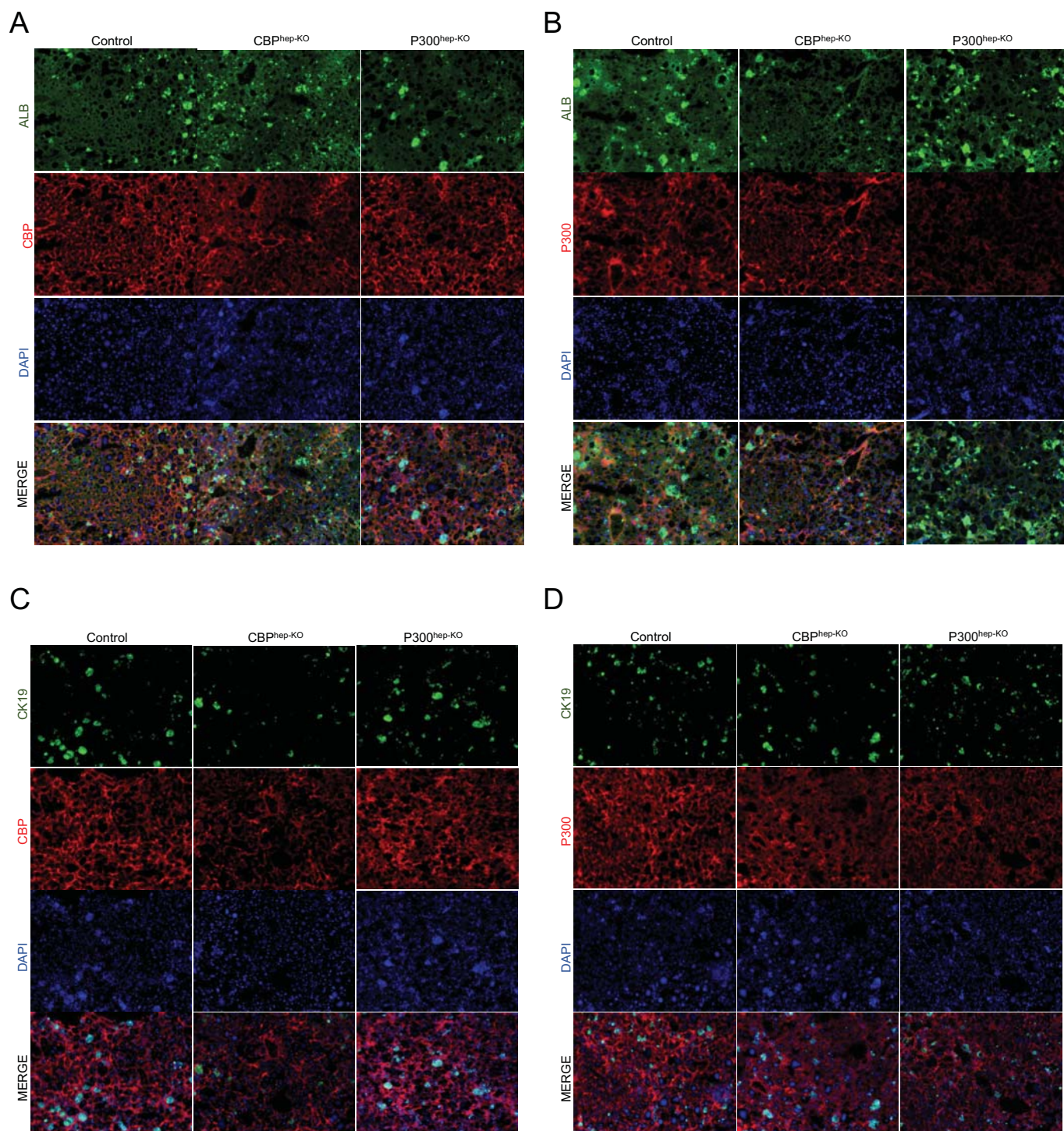

Supplementary Figure S2

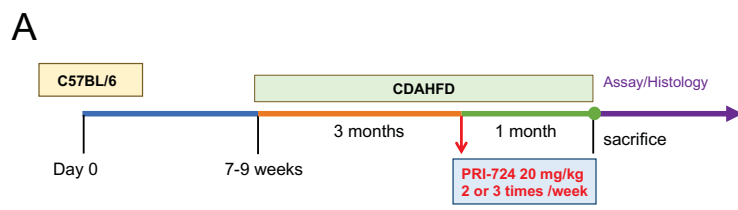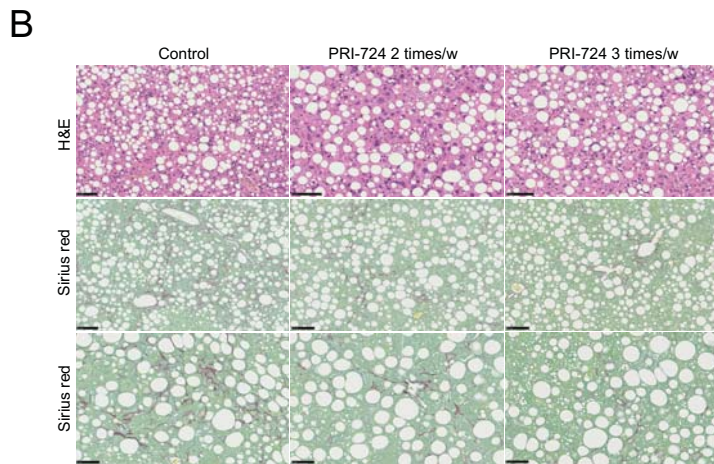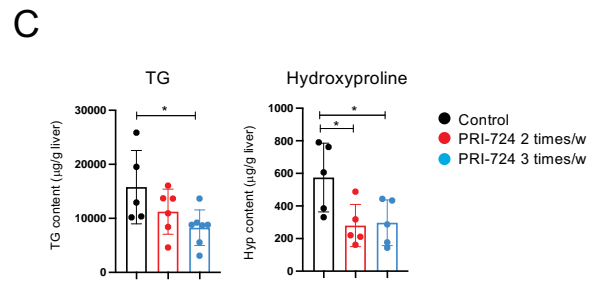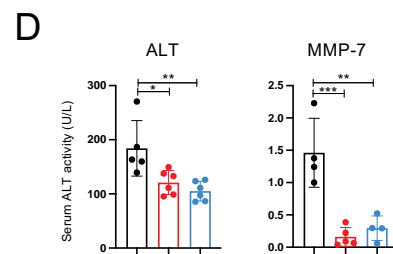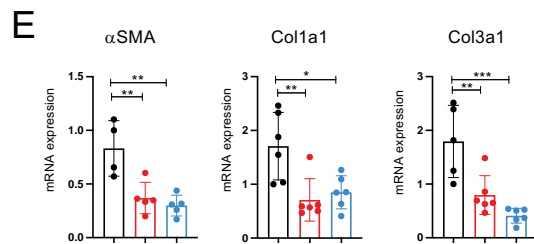

Supplementary Figure S3

Supplement: Supplementary file 1 — Figure S1: fsb271685‐sup‐0001‐FigureS1‐S3.pdf. [file FSB2-40-e71685-s001.pdf]
